# Supplementary material for: Engaging primary care professionals in suicide prevention: A qualitative study
Source: PLoS One. 2020 Nov 30;15(11):e0242540. doi: 10.1371/journal.pone.0242540 (PMC7704003; doi:10.1371/journal.pone.0242540)
Supplement: S1 Material — (DOCX) [file pone.0242540.s001.docx]

# S1 File. Topic list

| **Topic** | **Example questions** |
| --- | --- |
| **Exploring suicidality** | - How and when do you generally explore suicidal feelings? - How do you feel about exploring/asking about suicidal feelings? - Are there patients with whom you find that easier or more difficult? When and why? |
| **Follow up Measures** | - How do you deal with patients who are suicidal? - What kind of follow up measures do you generally take? - Are there any barriers or facilitators that you come across? What kind? - How would you describe the collaboration with other institutions such as MHC? |
| **SUPRANET intervention** | - What do you remember from the suicide prevention intervention that was offered in the context of SUPRANET? - Which of the elements have you participated in / were you involved in? - Which of the elements were most valuable / useful to you and why? - What did you think of the other elements? - Do you feel like you have gained something from it? What? - Are there aspects that you now do or look at differently? |
| **Suggestions for improvement** | - What have you missed in the intervention? - What could we improve about the intervention? |
| **Other pillars of supranet** | - Besides the intervention for PCPs, there were also other SUPRANET related activities in your region. What have you noticed of this? What did you think of these? |
| **Participating in the extended training package** | - Why did you decide to participate? What were your expectations? - Were there any incentives that facilitated or issues that complicated this decision? (e.g. accreditation, time or money)? What would be reasons not to participate? - Do you think it is important that more GP practices should participate? If so, what would be an effective manner to encourage them? |
| **Needs and other** | - Aside from the intervention, is there anything else that you need in order to engage in suicide prevention more effectively? - Is there anything else that you want to share? |
