# Supplementary material for: Engaging primary care professionals in suicide prevention: A qualitative study
Source: PLoS One. 2020 Nov 30;15(11):e0242540. doi: 10.1371/journal.pone.0242540 (PMC7704003; doi:10.1371/journal.pone.0242540)
Supplement: Attachment — Submitted filename: Response to reviewers.docx [file pone.0242540.s002.docx]

Dear Dr. Elzinga,

Thank you for submitting your manuscript to PLOS ONE. I have recently taken over this manuscript as Academic Editor and have had the opportunity to review the comments provided by the expert reviewer that the prior Academic Editor invited. I have also had a chance to review your paper as well. As you will see below, the reviewer had a generally positive opinion of the paper but had concerns that are likely addressable. Accordingly, I am inviting you to revise and resubmit your manuscript. Please do know that this is no guarantee of ultimate acceptance and I will send the revised version of this manuscript back to the original reviewer for comments again.

Although all of the reviewer's comments are important to address, there are a few comments in particular that I'd like you to pay attention to. Specifically, it would be good to add more detail on (a) the background of how PCPs are trained in suicide screening/prevention/intervention and (b) what the specific suicide screening/prevention/intervention programs that are implemented are. Additionally, as the reviewer notes, there are existing research frameworks where your study fits well (e.g,. The Consolidated Framework for Implementation Research) and it would be good to discuss this in your introduction.

Please submit your revised manuscript by Jul 18 2020 11:59PM. If you will need more time than this to complete your revisions, please reply to this message or contact the journal office at [plosone@plos.org](mailto:plosone@plos.org). When you're ready to submit your revision, log on to <https://www.editorialmanager.com/pone/> and select the 'Submissions Needing Revision' folder to locate your manuscript file.

Please include the following items when submitting your revised manuscript:

- A rebuttal letter that responds to each point raised by the academic editor and reviewer(s). You should upload this letter as a separate file labeled 'Response to Reviewers'.
- A marked-up copy of your manuscript that highlights changes made to the original version. You should upload this as a separate file labeled 'Revised Manuscript with Track Changes'.
- An unmarked version of your revised paper without tracked changes. You should upload this as a separate file labeled 'Manuscript'.

If you would like to make changes to your financial disclosure, please include your updated statement in your cover letter. Guidelines for resubmitting your figure files are available below the reviewer comments at the end of this letter.

If applicable, we recommend that you deposit your laboratory protocols in protocols io to enhance the reproducibility of your results. Protocols.io assigns your protocol its own identifier (DOI) so that it can be cited independently in the future. For instructions see: <http://journals.plos.org/plosone/s/submission-guidelines#loc-laboratory-protocols>

We look forward to receiving your revised manuscript.

Kind regards,

Evan M Kleiman
Academic Editor
PLOS ONE

*Dear dr. Evan Kleiman,*

*Thank you for providing the opportunity to revise the manuscript and considering it for publication in PLOS ONE. We believe that the revisions have improved the manuscript considerably. We have addressed your comments below using italic script.*

Journal Requirements:

When submitting your revision, we need you to address these additional requirements.

1. Please ensure that your manuscript meets PLOS ONE's style requirements, including those for file naming. The PLOS ONE style templates can be found at

<https://journals.plos.org/plosone/s/file?id=wjVg/PLOSOne_formatting_sample_main_body.pdf> and

<https://journals.plos.org/plosone/s/file?id=ba62/PLOSOne_formatting_sample_title_authors_affiliations.pdf>

*Thank you, I have adjusted the manuscript according to the style requirements.*

2. For qualitative studies, PLOS ONE suggests consulting the COREQ guidelines: <http://intqhc.oxfordjournals.org/content/19/6/349> to ensure that all relevant information is provided (in this case we would appreciate more information about: the number  and training of interviewers; how participants were selected; if bias issues were considered.

*Thank you for this comment. We have carefully read the COREQ guidelines and added information about the number and training of interviewers, participant selection and if biases were considered.*

*Line 126-128: “The interviews were conducted by one researcher (EE), who had completed certified training in qualitative research methods including interview skills, in the period October 2018 to March 2019.”*

*Line 105-110: “We used convenience sampling and the snowball technique to recruit study participants from the PCPs within the SUPRANET regions who completed the suicide prevention training. We reached out to PCPs directly or to contact persons in GP practices via email and by phone. In regions where implementation was lagging, i.e., where no PCPs were being trained before the study deadline, professionals who were involved with the implementation of the SUPRANET intervention were approached for an interview.”*

*Line 471-476: “Since participation in the intervention and evaluation study was voluntary, it is likely that especially PCPs with a special interest in suicide prevention predominated among those who signed up. Most of the PCPs of this study indeed reported they had a special interest or experience in suicidality or mental health issues, for example, by having worked in a MHC institution. This inherent bias in the sampling method means that the outcomes of the study may not be generalizable to the entire PCP population.”*

3. Please include the registration number for the clinical trial referenced in the manuscript.

*We have not referred to a clinical trial in this manuscript.*

4. In your Data Availability statement, you have not specified where the minimal data set underlying the results described in your manuscript can be found. PLOS defines a study's minimal data set as the underlying data used to reach the conclusions drawn in the manuscript and any additional data required to replicate the reported study findings in their entirety. All PLOS journals require that the minimal data set be made fully available. For more information about our data policy, please see <http://journals.plos.org/plosone/s/data-availability>.

Upon re-submitting your revised manuscript, please upload your study’s minimal underlying data set as either Supporting Information files or to a stable, public repository and include the relevant URLs, DOIs, or accession numbers within your revised cover letter. For a list of acceptable repositories, please see <http://journals.plos.org/plosone/s/data-availability#loc-recommended-repositories>. Any potentially identifying patient information must be fully anonymized.

Important: If there are ethical or legal restrictions to sharing your data publicly, please explain these restrictions in detail. Please see our guidelines for more information on what we consider unacceptable restrictions to publicly sharing data: <http://journals.plos.org/plosone/s/data-availability#loc-unacceptable-data-access-restrictions>. Note that it is not acceptable for the authors to be the sole named individuals responsible for ensuring data access.

*The paper includes anonymized quotes of the interviewees to support the results. It is not possible to make the full transcripts of the interviews publicly available. The interviewees have not given consent for this, and they may contain sensitive or identifiable information. The transcripts are archived on a secured server of 113 Suicide Prevention. Anonymized excerpts of transcripts are available upon request, which can be made via email to the corresponding author (Elke Elzinga;* [*e.elzinga@113.nl*](mailto:e.elzinga@113.nl)*) or to the research department of 113 Suicide Prevention (*[*onderzoek@113.nl*](mailto:onderzoek@113.nl)*).*

We will update your Data Availability statement to reflect the information you provide in your cover letter.

5. Please include a separate caption for each figure in your manuscript.

*We have included captions for all tables and excluded the figure from the manuscript.*

[Note: HTML markup is below. Please do not edit.]

**Reviewers' comments:**

Reviewer's Responses to Questions

Comments to the Author

1. Is the manuscript technically sound, and do the data support the conclusions?

The manuscript must describe a technically sound piece of scientific research with data that supports the conclusions. Experiments must have been conducted rigorously, with appropriate controls, replication, and sample sizes. The conclusions must be drawn appropriately based on the data presented.

Reviewer #1: Partly

 2. Has the statistical analysis been performed appropriately and rigorously?

Reviewer #1: Yes

3. Have the authors made all data underlying the findings in their manuscript fully available?

The PLOS Data policy requires authors to make all data underlying the findings described in their manuscript fully available without restriction, with rare exception (please refer to the Data Availability Statement in the manuscript PDF file). The data should be provided as part of the manuscript or its supporting information, or deposited to a public repository. For example, in addition to summary statistics, the data points behind means, medians and variance measures should be available. If there are restrictions on publicly sharing data—e.g. participant privacy or use of data from a third party—those must be specified.

Reviewer #1: No

4. Is the manuscript presented in an intelligible fashion and written in standard English?

PLOS ONE does not copyedit accepted manuscripts, so the language in submitted articles must be clear, correct, and unambiguous. Any typographical or grammatical errors should be corrected at revision, so please note any specific errors here.

Reviewer #1: No

5. Review Comments to the Author

Please use the space provided to explain your answers to the questions above. You may also include additional comments for the author, including concerns about dual publication, research ethics, or publication ethics. (Please upload your review as an attachment if it exceeds 20,000 characters)

Reviewer #1: Thank you for the opportunity to review this manuscript. This paper presents the results of qualitative interviews with 21 individuals involved in the implementation of a primary care based suicide prevention program, a timely and important topic. I have several concerns about the manuscript in its current form, detailed below, that I think would be helpful for the authors to address.

*Dear reviewer,*

*Thank you for you carefully formulated comments. We have addressed them in the manuscript and in this letter, using italic script.*

While the manuscript is generally well written, additional proofreading by a native English speaker would be helpful.

*Thank you for your suggestion, the manuscript is now proofread by a professional language bureau.*

Perhaps it is just a problem with the proof, but the text in Box 1 appears cut off.

*Thank you for noticing, the box was indeed cut off. We have deleted the box and incorporated it in the introduction.*

*Line 65-69: “Since 2007, GPs can also refer patients to the Mental Health Nurse (MHN), a role introduced to cope with the increasing demand for mental health issues in primary care [21]. MHNs include professionals with a nursing or psychology background who can provide brief therapy sessions to patients within primary care [10]*.”

It might be helpful to describe the suicide screening/prevention/intervention practices being implemented as the “interventions” and the additional training and support being offered to the clinicians as the “implementation strategies.” It would also be helpful to understand the intensity of training and supports provided to PCPs. Powell et al’s (2015) refined compilation of implementation strategies paper and Proctor et al.’s (2013) paper on specifying and reporting implementation strategies, both published in Implementation Science, may be helpful in these regards. I think this approach would improve clarity throughout.

*Thank you for this suggestion. We have clarified the terms by referring to “intervention” with regard to the entire package, including the suicide prevention training, continuity of care element, module on suicide and medication, suicide prevention guide, flyers and posters and checklist (see table 1 below), and by referring to “implementation strategies” with regard to offering the intervention to the clinicians all over the manuscript. We have also adjusted the table and added extra information about what these elements entail and described the intensity of the elements.*

*Line 102:*

Table 1. Various elements of the SUPRANET intervention for PCPs

| *Suicide prevention training* | *The training consists of a theoretical section covering the epidemiology, behaviour, and process of suicide, the Chronological Assessment of Suicidal Events (CASE) methodology, and treatment and referral. The tuition is interspersed with interactive exercises focused on making contact with feelings of despair, involving relatives, and diagnosis. The training is provided by experienced trainers from the Dutch College of General Practitioners and is accredited for four hours.* |
| --- | --- |
| *Continuity of care* | *CPs are encouraged to arrange meetings with organizations in the regional chain of care for suicidal patients (e.g., MHC institutions, crisis services, or emergency rooms) to discuss issues and to enter into agreements to improve the continuity of care.* |
| *Module on suicide and medication* | *A two-hour (accredited) individual e-learning session or a pharmaceutical therapeutic audit completed with colleagues takes place on the subject of the role of medication in suicide prevention.* |
| *Suicide prevention guide* | *The suicide prevention guide contains a summary of the most important information from the suicide prevention training and the multidisciplinary guideline for the diagnosis and treatment of suicidal behaviour including a triage tool to help in referrals of suicidal patients.* |
| *Flyers and posters* | *Flyers and posters are aimed at patients and relatives, to encourage them to talk about and seek help for suicidal feelings.* |
| *Checklist* | *The checklist can be completed after consultations with (possible) suicidal patients. The checklist includes items such as assessment of suicidal feelings, rumination, and the concreteness of suicidal plans.* |

The authors are encouraged to consider making the “topic list” used to guide interviews available as supplemental to the paper. What was the rationale for this approach versus a more traditional semi-structured qualitative interview guide? A semi-structured guide has several advantages including ensuring uniform inclusion and sequencing of questions.

*Thank you for this suggestion. We have added the topic list in the supplementary files (S1 File. Topic list) and explained the reasons for this approach in the method section.*

*Line 114-119: “A topic list was used to guide the semi-structured interviews (See S1. Topic list). This instrument consists of a list of topics and prompts based on literature [24–26] and discussed within the research group (AK, DB, RG, EE). The rationale for this approach is that it is best suited to capture the perceptions and experiences of participants [27]. Although we mostly addressed the topics in the given order, if required, we could discuss them in another order, and it leaves space for participants to talk freely and bring up topics or issues themselves”*

If there is any additional demographic information about participants please consider including it, for example participant race, ethnicity, mean age, mean number of years in practice, and information about the types of practices (community clinics vs. academically affiliated centers for example).

*Unfortunately, there is not. We only have information regarding function and gender (and region, but that cannot be published due to privacy restrictions).*

Some of the content of the results seems to report perceptions or suggestions made by one PCP. While it is certainly appropriate to provide illustrative quotations from participants, the reader can be much more confident in the trustworthiness of overall themes that emerged based on the perspectives of many of the interviewees and not individual interviewee perspectives. It may be that this is simply an issue with how the results were presented in the paper and not the actual coding process.

*Thank you for your suggestion. The results are not based upon the data from one PCP, but on all 21 participants. We have included numbers of the interviewees to the quotes, hopefully this clarifies that the comments originated from different participants.*

What is 113 referring to in the Results? Is this masking a person or program or an actual name of a program?

*113 refers to 113 Suicide prevention: the initiator of SUPRANET. We have now addressed this (more clearly) in the method section.*

*Line 85-86: “SUPRANET was initiated by 113 Suicide Prevention, the Netherlands national suicide prevention centre, as part of the national agenda for suicide prevention commissioned by the Ministry of Health”.*

Consider grounding this work in an established implementation framework, like the Consolidated Framework for Implementation Research (CFIR). Most of the themes fit within established constructs from frameworks like CFIR and organizing and presenting them in this way could be useful.

*We have carefully read the paper about the CFIR and it seems to provide a very relevant framework for when performing an implementation study. In this evaluation study, where we focused on the perceptions and experiences of PCPs with the intervention, we did not select a framework to guide the data collection and analysis procedure upfront. Therefore, we chose to discuss the CFIR it in the discussion.*

*Lines 407-426: “Although we did not select a framework a priori, using a framework, such as Damschroder and colleagues’ Consolidated Framework for Implementation Research (CFIR), may aid in the evaluation of complex interventions such as SUPRANET [34]. The CFIR consists of five domains (intervention characteristics, outer setting, inner setting, characteristics of individuals, and process) that interact in various ways and determine the effectiveness of implementation. When applying the CFIR retrospectively, we were able to establish some factors that may have played a crucial role in the effectiveness of the intervention. The suicide prevention training can be seen as the “core component” with an “adaptable periphery” consisting of the other elements and its flexible delivery. According to the CFIR, this facilitates implementation and thus influences effectiveness. Further, peer pressure from other PCPs, perceived patients’ needs, and time constraints can be identified as factors from the outer setting that influence participation. Factors from the inner setting include a positive climate for implementation within the GP practices, shown for example by PCPs who wanted to participate because of experiences with a patient’s suicide (tension for change), because they wanted to develop their skills (positive learning climate), or because of the perceived urgency of the topic (relative priority). This study also indicated that some individuals, notably key PCPs, played an important role in the implementation of the intervention. The same applies in reverse; implementation in certain regions may have lagged behind others because no such individuals were driving the initiative. Although we could apply this framework retrospectively, more or other factors may have come up when applying it from the beginning. Therefore, future evaluation studies are recommended to use this or another framework to guide the evaluation from the start.”*

- An integrated coding approach (see Bradley) that combines inductive and deductive coding could offer advantages to solely utilizing grounded theory here.

*In their article Bradley et al 2007 focus on qualitative data analysis that has as a goal the generation of taxonomy, themes, and theory germane to health services research. Because of the explorative nature of our research we conducted a grounded theory approach. Our approach was inductive and data-driven, focused upon identifying and discussing the salient themes repeated across and within transcripts. It involves coding and the generation (and interpretation) of broader patterns in data. Our choice is therefore prompted by carrying out the analysis from this point of view.*

- It could be helpful to review the CFIR implementation process constructs in particular to see if those may be useful for organizing the presentation of the results around providers experience with implementation.

*Thank you for your suggestion. Even though evaluation the implementation process was not the main goal of the paper, implementation-related themes did emerge from the data which we addressed in the chapter ‘implementation strategies’ (lines 165-207). Further, we discussed the application of the CFIR framework onto our results in the discussion. From the implementation construct, we were especially able to establish the role of certain individuals (key PCPs) during the implementation process.*

*Line 421-425: “This study also indicated that some individuals, notably key PCPs, played an important role in the implementation of the intervention. The same applies in reverse; implementation in certain regions may have lagged behind others because no such individuals were driving the initiative. Although we could apply this framework retrospectively, more or other factors may have come up when applying it from the beginning.”*

- Another suggestion would be to specifically pull out barriers, facilitators, and suggested strategies/changes and present as their own themes. Presently these are presented within other themes.

*Thank you for this suggestion. However, the rationale of this study was to provide insights into the perceptions and experiences of PCPs with the intervention and suicide prevention in general practice, which will ultimately be used to enhance the intervention and to engage PCPs more effectively in suicide prevention. The topic list that we used in this study was based on this rationale. Although barriers and facilitators emerged, we did not set up this study to focus on those specifically. It should be noted that the barriers and facilitators that are being described in the present study emerged within the context of the themes and are non-exhaustive. From this perspective, we think the chosen structure into the main themes is most suitable.*

Finally, I see that e-learning is mentioned in the discussion as a strategy that was suggested by providers, but I don’t recall seeing that presented in the results. I suggest ensuring that all themes commented on in the discussion are reported in the results.

*Thank you, we have described this in the result section:*

*Line 238-239: “Other suggestions included shortening the training or splitting it up into two parts: online individual training complemented with training in a group to practice communication skills.*

6. PLOS authors have the option to publish the peer review history of their article (what does this mean?). If published, this will include your full peer review and any attached files.

If you choose “no”, your identity will remain anonymous but your review may still be made public.

Do you want your identity to be public for this peer review? For information about this choice, including consent withdrawal, please see our Privacy Policy.

Reviewer #1: No

*Once again, thank you for your comments. We believe that with these revisions the manuscript has improved a lot.*
