# Supplementary material for: Engaging primary care professionals in suicide prevention: A qualitative study
Source: PLoS One. 2020 Nov 30;15(11):e0242540. doi: 10.1371/journal.pone.0242540 (PMC7704003; doi:10.1371/journal.pone.0242540)
Supplement: Attachment — Submitted filename: Rebuttal letter.docx [file pone.0242540.s003.docx]

*Dear editor and reviewer,*

*Thank you for the opportunity to revise the manuscript once more. We have discussed your comments thoroughly within our research team and addressed these in the manuscript. Below, we will provide an answer to the issues one-by-one:*

Reviewer #1: Thank you for the opportunity to re-review this manuscript. I appreciate the detailed rationale provided in the authors’ response to reviews. There are a few remaining issues that warrant consideration.

Minor point- Table 2. I do not think this level of detail about participants is necessary, rather I would suggest reporting in aggregate the n (%) of participants who are male, GPs etc.

*Thank you for your comment, we have adjusted the table (line 149).*

There remains some confusion with respect to the intervention vs. implementation strategies. In the way these terms are typically used, the interventions are the evidence based suicide prevention strategies that the PCPs use with their patients. The implementation strategies are the strategies employed in order to get PCPs to implement EBPs for suicide prevention, such as training, consultation, facilitation etc.
Geoff Curran has a paper on the topic that very clearly describes the distinction: Curran, G.M. Implementation science made too simple: a teaching tool. Implement Sci Commun 1, 27 (2020). <https://doi.org/10.1186/s43058-020-00001-z>

Specifically, it would be helpful to clearly distinguish participants’ reactions to the SUPRANET training/supports (the implementation strategies) vs. their reactions to implementing EBPs (evidence-based practices) for suicide prevention (the intervention).

*Thanks for clearing up the confusion. We have changed this throughout the manuscript; SUPRANET is now described as implementation strategy to help PCPs applying suicide prevention practices. See for example the altered aim on line 75-78:*

*“Using a qualitative design, we conducted this implementation study to evaluate to what extent SUPRANET was helpful in supporting PCPs to apply suicide prevention practices. These insights will be used to engage PCPs more effectively in suicide prevention by improving the use of SUPRANET.”*

I think the way the CFIR is integrated at present is confusing. I understand you do not wish to retrospectively apply CFIR to your data and that is fine, however if that is the case then I would not attempt to do that in the Discussion as you did. Instead, I would suggest removing CFIR from the Discussion and incorporating it into the Introduction. I do believe that this is in fact an implementation study- you are studying an effort to train providers to implement suicide prevention practices, exploring PCPs reactions and utilization of suicide prevention practices, etc. In contrast, an intervention study would typically involve studying the impact of suicide prevention practices on patients. Again, Geoff Curran’s paper may be helpful clarification.
Given this, CFIR seems quite relevant. In the Introduction I would suggest you introduce the CFIR as a comprehensive framework that describes the relevant contextual factors that have been shown to be important to consider when planning for the implementation of a health service intervention (e.g., suicide prevention EBPs). Then, I would briefly describe the 5 main CFIR constructs and delineate which you focused on in the present project. For example, Characteristics of the Intervention (e.g., the characteristics of suicide prevention EBPs that may impact their use in a particular setting or by particular providers), Outer Setting (e.g., external factors such as political or policy factors related to suicide prevention, patient populations served by the PCPs), Inner Setting (e.g., what is it about the way clinics are structured or ways in which workflows or availability of behavioral health consultant impacts practice?), Characteristics of the Individuals implementing suicide EBPs (e.g., PCP attitudes about suicide prevention, self-efficacy to implement suicide EBPs) and Implementation Process (e.g., what was the experience of receiving training through SUPRANET like).
I hope this helps to clarify some of my previous comments from the initial submission.

*Thanks for your explanation. We have now introduced the CFIR in the introduction, explained how we apply the model in the method-section and we have organized the results according to the CFIR structure. In the discussion we present the most important findings according to the CFIR (see below).*

*Introduction (line 70-75)*

*“An often-used framework to address implementation challenges is the Consolidated Framework for Implementation Research (CFIR). This comprehensive framework describes factors that are important in implementing and evaluating complex interventions. It consists of five domains (characteristics of the intervention, outer setting, inner setting, characteristics of individuals, and process of implementation) which interact and determine effectiveness of implementation together [20].”*

*Methods (line 83-87)*

*“The CFIR model was used to structure and organize the data. In the present study, intervention characteristics refers to PCPs’ experiences with applying suicide prevention practices such as exploring suicidal feelings. Outer setting refers to the level of collaboration with MHC and in inner setting we discuss the role of MHNs with regard to suicide prevention. The process of implementation describes how the implementation strategy, SUPRANET, was received by PCPs.”*

*Results (line 144-331)*

*see manuscript*

*Discussion (line 335-344)*

*“The CFIR was used to structure the data and to gain insights in facilitating and limiting factors of SUPRANET. All but one domain (individual characteristics) from the CFIR were explicitly addressed. Since this study was based on PCPs’ perceptions and experiences, individual characteristics are intertwined with other domains, particular with intervention characteristics. This study found that there were important barriers to the assessment of suicide risk in patients (intervention characteristics) because of PCPs’ self-perceived incompetence, burdensomeness of suicide and their lack of time and workload. The relationship with MHC was an important limiting factor from the outer setting and with regard to inner setting, MHSS had a positive influence on implementation. Although there were some difficulties in the process of implementation, SUPRANET itself was perceived as useful strategy to improve suicide prevention practices. “*
